# AtSEC22 Regulates Cell Morphogenesis via Affecting Cytoskeleton Organization and Stabilities

**DOI:** 10.3389/fpls.2021.635732

**Published:** 2021-06-04

**Authors:** Li Guan, Shurui Yang, Shenglin Li, Yu Liu, Yuqi Liu, Yi Yang, Guochen Qin, Haihai Wang, Tao Wu, Zhigang Wang, Xianzhong Feng, Yongrui Wu, Jian-Kang Zhu, Xugang Li, Lixin Li

**Affiliations:** ^1^Key Laboratory of Saline-Alkali Vegetation Ecology Restoration, Ministry of Education, College of Life Sciences, Northeast Forestry University, Harbin, China; ^2^State Key Laboratory of Crop Biology, College of Life Sciences, Shandong Agricultural University, Tai’an, China; ^3^Shanghai Center for Plant Stress Biology, CAS Center for Excellence in Molecular Plant Sciences, Chinese Academy of Sciences, Shanghai, China; ^4^National Key Laboratory of Plant Molecular Genetics, CAS Center for Excellence in Molecular Plant Sciences, Institute of Plant Physiology and Ecology, Shanghai Institutes for Biological Sciences, Chinese Academy of Sciences, Shanghai, China; ^5^Key Laboratory of Soybean Molecular Design Breeding, Northeast Institute of Geography and Agroecology, Chinese Academy of Sciences, Changchun, China; ^6^School of Life Sciences and Agriculture and Forestry, Qiqihar University, Qiqihar, China

**Keywords:** AtSEC22, membrane trafficking, cytoskeleton dynamics, cell morphogenesis, plant growth and development

## Abstract

The plant cytoskeleton forms a stereoscopic network that regulates cell morphogenesis. The cytoskeleton also provides tracks for trafficking of vesicles to the target membrane. Fusion of vesicles with the target membrane is promoted by SNARE proteins, etc. The vesicle-SNARE, Sec22, regulates membrane trafficking between the ER and Golgi in yeast and mammals. *Arabidopsis* AtSEC22 might also regulate early secretion and is essential for gametophyte development. However, the role of AtSEC22 in plant development is unclear. To clarify the role of AtSEC22 in the regulation of plant development, we isolated an *AtSEC22* knock-down mutant, *atsec22-4*, and found that cell morphogenesis and development were seriously disturbed. *atsec22-4* exhibited shorter primary roots (PRs), dwarf plants, and partial abortion. More interestingly, the *atsec22-4* mutant had less trichomes with altered morphology, irregular stomata, and pavement cells, suggesting that cell morphogenesis was perturbed. Further analyses revealed that in *atsec22-4*, vesicle trafficking was blocked, resulting in the trapping of proteins in the ER and collapse of structures of the ER and Golgi apparatus. Furthermore, AtSEC22 defects resulted in impaired organization and stability of the cytoskeleton in *atsec22-4*. Our findings revealed essential roles of AtSEC22 in membrane trafficking and cytoskeleton dynamics during plant development.

## Introduction

Plant cell shape formation is crucial for development and morphogenesis, which are regulated by cytoskeletal dynamics (Fu et al., 2005; Panteris and Galatis, 2005; Kotzer and Wasteneys, 2006; Zhang et al., 2011; Szymanski, 2014; Armour et al., 2015; Chen et al., 2016; Christopher and Arun, 2018). The cytoskeleton, comprised of actin filaments (AFs) and microtubules (MTs), forms a three-dimensional intracellular network that provides tracks for cellular transport, including organelles, vesicles, protein complexes, and macromolecules (Wasteneys and Yang, 2004; Hussey et al., 2006; Akhmanova and Hammer, 2010). Cortical MTs control anisotropic cell expansion by directing the deposition pattern of cellulose microfibrils in the root and hypocotyl (Fu et al., 2005; Takatani et al., 2015). The cell shape is controlled by MT dynamics modulated by microtubule binding proteins (MAPs) and by the coordination between cortical MTs and AFs (Burk et al., 2001; Fu et al., 2005; Panteris and Galatis, 2005; Ambrose et al., 2007, 2011; Kirik et al., 2007; Wang et al., 2007; Zhang et al., 2013). Cortical AFs provide endomembrane guidance and structural support as well as a driving force by forming dynamic fringe structures at the leading edge during polarized cell growth (Vidali et al., 2009). The particular shape of a cell is important for its function and environmental adaptation (Robert, 2013). Trichomes, guard cells, and pavement cells are highly specialized cell types generated from the leaf epidermal layer (Vöfély et al., 2019). The *Arabidopsis* trichome is a unicellular structure with usually three branches; MTs are responsible for trichome initiation and branching, and AFs control the shape (Sambade et al., 2014; Chen et al., 2016). It has been shown in detail that AFs and MTs mediate vesicle trafficking in plants (Žárský et al., 2009; Mooren et al., 2012; Idilli et al., 2013; Kong et al., 2015).

In eukaryote, vesicle trafficking mediates the delivery of proteins to their destinations and maintains the endomembrane system. Most of the secreted proteins are synthesized in the rough ER and transported to the target compartments. Vesicle transport involves vesicle budding from the donor membrane, movement along the cytoskeleton, and fusion with the target membrane (Palade, 1975; Rothman, 2014). Cargo recruitment and vesicle formation in the ER are mediated by the GTPase SAR1, the guanine-nucleotide exchange factor (GEF) Sec12, and the coat protein II (COPII) complex (Tang et al., 2005; Gürkan et al., 2006). ER-derived COPII vesicles fuse to the cis-Golgi cisterna and release the cargoes (Kurokawa et al., 2014; Lee et al., 2016). Conversely, Golgi-derived COPI vesicles fuse to the ER membrane to retrieve the membrane component and ER-retention proteins for recycling (Yu et al., 2012; Brandizzi and Barlowe, 2013; Spang, 2013; Dodonova et al., 2015). Membrane fusion is mediated by conserved proteins including Rab GTPases, tethers, and soluble N-ethylmalemide-sensitive factor attachment protein receptors (SNAREs), which contain SNARE motifs (Söllner et al., 1993; Hanson et al., 1997; Bonifacino and Glick, 2004; Lipka et al., 2007; Saito and Ueda, 2009). Membrane fusion is conducted by the formation of a trans-SNARE complex composed of one vesicle membrane-SNARE (v-SNARE) and two or three target membrane-SNAREs (t-SNAREs) (Söllner et al., 1993; Li et al., 2016; Bruinsma et al., 2018). In yeast and mammalian cells, v-SNARE Sec22 mediates anterograde and retrograde transport between the ER and the Golgi complex (Newman et al., 1990; Hardwick and Pelham, 1992; Flanagan et al., 2015; Li et al., 2015; Zhao et al., 2015; Lee et al., 2016). In yeast, in anterograde transport, fusion of ER-derived COPII vesicles with Golgi cisterna requires Sec22p, which forms a SNARE complex with the cis-Golgi localized t-SNAREs, Sed5p, Bos1p, and Bet1p. Conversely, in retrograde transport, Sec22p is combined with ER-localized Ufe1p, Slt1p, and Sec20p (Liu and Barlowe, 2002; Burri et al., 2003; Morsomme et al., 2003; Brandizzi and Barlowe, 2013; Spang, 2013; Dodonova et al., 2015). COPI vesicles are implicated in the quick recycling of Sec22 from the Golgi to the ER under salt stress (Letourneur et al., 1994; Ossipov et al., 1999). In tobacco leaves, transiently overexpressed *Arabidopsis* SEC22 was localized on the ER and Golgi apparatus and induced collapse of Golgi membrane proteins and redistribution into the ER, suggesting that it functions in the early secretion pathway (Chatre et al., 2005). El-Kasmi et al. have reported that AtSEC22 is essential for gametophyte development and Golgi integrity. Loss of *AtSEC22* has been shown to result in Golgi fragmentation and pollen lethality (El-Kasmi et al., 2011). Moreover, Sec22p/SEC22 specifically impacted Cs^+^ accumulation in yeast and plants. Sec22p has also been shown to enhance non-selective cation deposition (Draxl et al., 2013). Cooperating with Sar1 and Memb11, Sec22 facilitates ER export of Phyl1.1 (Phytolongin), which is important for vesicle formation and fusion in plants (de Marcos Lousa et al., 2016).

In order to further investigate SEC22 function in plant development, we isolated an *AtSEC22* knockdown mutant, *atsec22-4*, which has developmental defects such as delayed germination, shorter primary roots (PRs), dwarf, and sterility. Interestingly, the morphology of trichomes, pavement cells, and stomata are altered in *atsec22-4*. Our investigation revealed that AtSEC22 interacts with the Golgi-resident syntaxin AtSYP32, to regulate vesicle transport between the ER and Golgi. Downregulation of *AtSEC22* blocked ER export. Furthermore, we found that cytoskeleton organization and stability were disrupted in *atsec22-4*, which resulted in impaired cell morphogenesis, stomata movement, and delayed development. Our findings illustrate the essential role of AtSEC22 in cell morphogenesis during plant development.

## Materials and Methods

### Plant Materials and Growth Conditions

*Arabidopsis thaliana* ecotype Col-0 was used as a wild-type plant. T-DNA-tagged lines were derived from Col-0. Homozygous plants were obtained by PCR screening using the insertion-specific primers shown in Supplementary Table 1. *Arabidopsis* seeds were surface-sterilized and sown either on soil or onto 0.8 or 1.2% agar with 1/2 Murashige and Skoog medium (PhytoTech) and 1% (w/v) sucrose. Plants were grown at 22°C under 16 h: 8 h light: dark cycles. *atsec22-4* (SAIL_736_F03), *atsyp32-1* (GABI-109A09), and *atsyp32-2* (GABI_920F05) were obtained from the *Arabidopsis* Biological Resource Center (ABRC) at Ohio State University. The fluorescence marker lines of *ST-GFP* (Boevink et al., 1998), *GFP-HDEL* (Matsushima et al., 2002), *pUBQ:ABD2-GFP* (Tian et al., 2015), *p35S:MBD-GFP* (Dyachok et al., 2014), *p35S:TUA6-GFP* (Eisinger W. et al., 2012), and *pPIN1:PIN1-GFP* (Benkova et al., 2003) were employed.

### Plasmid Construction

An *AtSEC22* cDNA fragment was amplified from Col-0 using the AtSEC22-specific primers *SEC22TOPO-F* and *SEC22TOPO-R* and ligated into *pENTR/D-TOPO* vectors (Invitrogen, Carlsbad, CA, United States). To generate TAP-tagged (containing 9xmyc myc) *AtSEC22*-expressing transgenic plants, *the AtSEC22* cDNA fragment was transferred from the *AtSEC22* entry clone to the destination vector *pNTAPa* (Rubio et al., 2005) by an LR reaction (Invitrogen). For *generating AtSEC22* RNAi plants, a 390 bp fragment of *AtSEC22* cDNA was amplified using the primers *Sec22RNAi-F* and *Sec22RNAi-R*, and cloned into *the pENTR/D-TOPO* vector and subsequently subcloned into the destination vector *pK7GWIWG2* by the LR reaction. To generate *pAtSEC22:gAtSEC22* complementation plants, the *AtSEC22* genomic sequence was amplified using the primers *Sec22comple-F* and *Sec22comple-R* and cloned into *the pDONR207* ENTRY vector (Invitrogen), and subsequently subcloned into the destination vector pMDC99 by the LR reaction. The primers used are listed in Supplementary Table 1.

### Anti-AtSEC22 Antibody Generation

To prepare the antigen, the AtSEC22 cytosolic fragment corresponding to 1–195 amino acids was amplified using the primers SEC22TOPO-F and SEC22TOPO-R, ligated into pENTR/D-TOPO vectors, and subsequently introduced into the pET32a vector (Novagen). Recombinant protein was expressed in the *Escherichia coli* BL-21 strain, purified with a HiTrap chelating column, and entrusted to PhytoAB Inc., to generate polyclonal antibodies. Immunoblot detected the AtSEC22 band using anti-AtSEC22 antibody from total extracts of Col-0 and *atsec22-4* seedlings.

### Immunoblotting

Dodecyl sulfate, sodium salt-Polyacrylamide gel electrophoresis (SDS-PAGE) and immunoblot analyses were performed as described previously (Li et al., 2006). Antibodies were diluted as follows: anti-AtSEC22, 1:500; anti-12S, 1:20,000; anti-2S3P, 1:5,000 (Li et al., 2006); anti-BiP (AS09 481, Agriser, Sweden), 1:2,500; anti-actin (AS13 2640, Agriser), 1:1,500; anti-tubulin A1 (At1g64740) (R0267-1a, Abiocode), 1:1,500; and MAP65-1 (0732B5, 9632B9, PhytoAB Inc.), 1:1,000, respectively. The secondary antibody was against rabbit IgG (ZB2301 and ZSGB-BIO), 1:5000. Immunoreactive signals were detected using an enhanced chemiluminescence detection system (LAS-4000 and FYJIFILM).

### Yeast Two-Hybrid Assay

For the yeast two-hybrid assay, *AtSYP81* plasmids were generated in a previous study (Li et al., 2006). The cytosolic regions of *AtSEC22*, *AtSYP31*, and *AtSYP32* were amplified using corresponding specific primers, *Sec22NdeI-F/Bam*HI*-R*, *SYP31/32NdeI-F*, and *SYP31/32BamHI-R*, respectively. Then these fragments were ligated into the *pEASY-Blunt* vector (TransGen, #CB101-01), respectively. After Sanger sequencing confirmation, the fragments were transferred into *pGADT7* or *pGBKT7* vectors, respectively. The paired constructs were introduced into *Saccharomyces cerevisiae* strain AH109 (Clontech) and selected on SD/-Leu/-Trp medium. The interactions were examined on SD/-Leu/-Trp/-His/-Ade medium.

### RNA Extraction and RT-PCR Analysis

Total RNA was isolated from seedlings of Col-0 and *atsec22-4* using the RNeasy kit (P4623, Tiangen, China). Total RNA (0.5–1 μg) was treated with DNase I (Invitrogen) to reduce genomic DNA contamination. Reverse transcription was performed using the All-in-One First Strand Synthesis MasterMix (NOVA). Semi-quantitative reverse transcription-polymerase chain reaction (RT-PCR) was performed according to the manufacturer’s instructions. *ACT2* was used as an endogenous control for RT-PCR and quantitative reverse transcription PCR (RT-qPCR). The specific primers are listed in Supplementary Table 1.

### Pull-Down Assay

Pull-down assays were performed as described previously (Li et al., 2013) using an iMACS epitope tag protein isolation kit (anti-c-myc, Miltenyi Biotec^1^). Two grams of two-week-old seedlings were used for each sample. The beads were sent for Shotgun liquid chromatography-tandem mass spectrometry (LC-MS/MS) analysis.

### Shotgun LC-MS/MS Analysis

LC-MS/MS analysis was performed as described previously (Qin et al., 2017), with modifications. Briefly, interacting proteins were eluted and reduced from beads by SDS elution buffer at 95°C for 5 min. Proteins were alkylated and digested on a centrifugal filter unit (10 kDa MWCO) using the filter-aided sample preparation (FASP) method. Peptides were analyzed by nanoAcquity ultra performance LC (Waters, Milford, MA, United States) and Orbitrap Fusion mass spectrometry (Thermo Fisher Scientific, Waltham, MA, United States). MS survey scan was performed by Orbitrap at a resolution of 60,000 over a m/z range of 350–1,800, and the top 20 precursor ions were selected for MS/MS measurements by HCD scans. Dynamic exclusion was enabled for 60 s. MS/MS raw data were searched against the database of The *Arabidopsis* Information Resource (TAIR10) using Mascot Daemon 2.5 (Matrix Science, London, United Kingdom). Carbamidomethylation of Cys was designated as a fixed modification. Deamidation of Asn or Gln and oxidation of Met were considered as variable modifications. Peptide assignments were filtered by an ion score cut-off of 15, and false discovery rate (FDR) of peptides was set up to less than 1%. The original data are presented in Supplementary Table 2.

### Chemical Treatments

For MT-depolymerization, 10 μM of oryzalin was applied to plant tissues for 10 min for hypocotyl and 20 min for leaf (Chen et al., 2016). For AF disruption, rosette leaves were incubated in 1 μM of LatB solution for 20 min (Konopka et al., 2008). Mocks were the same solvent without the reagents. For FM4-64 staining, plant tissues were incubated for 5 min in 4 μM of FM4-64 solution (Rigal et al., 2015).

### Confocal Microscopy

Fluorescent images were obtained using a point scanning confocal microscope (Leica TCS SP8). Confocal imaging was preset for GFP (with Ex:488 nm, Em:500–550 nm) or for RFP and DsRED (with Ex:543 nm, Em: 580–640 nm).

### Scanning Electron Microscope and Transmission Electron Microscopy Analysis

The 3rd or 4th rosette leaves from seven-week-old plants were collected and analyzed by Scanning Electron Microscope (SEM). Trichomes were observed using a JSM-6610LV Emission SEM (Hitachi). Transmission electron microscopy (TEM) observations were performed as described previously (Shimada et al., 2006) with leaves from two-week-old plants. The sections were observed and photographed using a transmission electron microscope H7700 (Hitachi).

### Accession Numbers

Gene and protein sequence data regarding this study can be found in the TAIR^2^ databases under the following accession numbers: *AtSEC22* (At1g11890), *AtSYP32* (At3g243500), *AtSYP31* (At5g05760), *AtSYP81* (At1g51740), *AtSec20* (At3g24315), and *MAP65-1* (At5g55230).

## Results

### *atsec22-4* Mutant Exhibits Serious Developmental Defects

**FIGURE 1:**
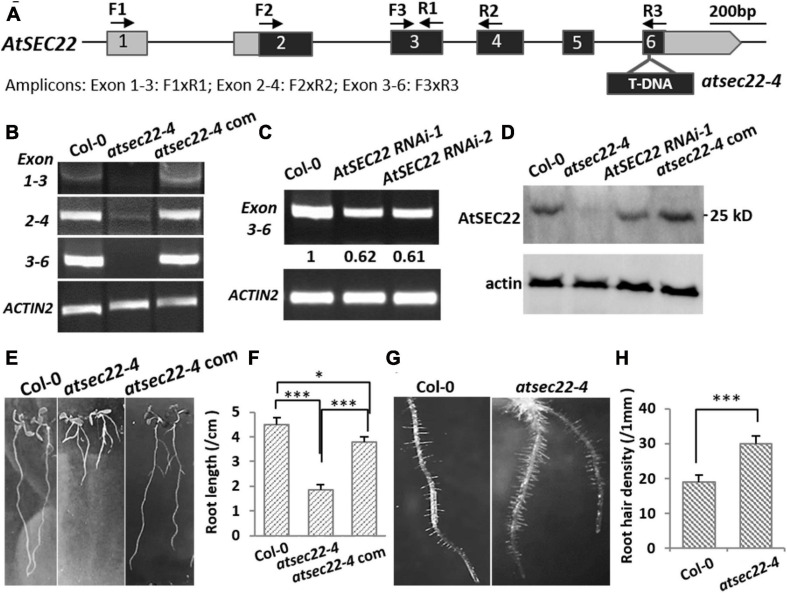
*AtSEC22* gene and defective mutants. **(A)** The structure of *AtSEC22* gene and the T-DNA insertion site in *atsec22-4*. Location of primers for RT-PCR analysis are indicated by arrows. Exons are indicated by boxes with numbers, and the solid lines indicate introns. **(B,C)** RT-PCR detection of expression level of *AtSEC22* with different combinations of primers in Col-0, *atsec22-4*, and *atsec22-4* complemented lines (*atsec22-4* com). *Actin2* was used as endogenous control. Exon 1-3: F1xR1; Exon 2-4: F2xR2; Exon 3-6: F3xR3. **(D)** Immunoblot was performed with ten-day-old seedlings of Col-0, *atsec22-4*, *AtSEC22* RNAi, and *atsec22-4* complemented lines using the anti-AtSEC22 antibody. **(E,F)** Ten-day-old seedlings of *atsec22-4* exhibited short primary roots and more adventitious roots. Panel **(F)** shows statistics of panel **(E)**. *n* ≥ 30, three independent experiments per sample. **(G,H)** Root hairs are denser in *atsec22-4.* Panel **(H)** shows statistics of panel **(G)**. *n* ≥ 10 roots. ***P < 0.001. Significance was evaluated by Student’s *t* test.

To explore the mechanisms underlying AtSEC22-mediated regulation of plant development, we isolated the *atsec22-4* mutant, in which T-DNA was inserted into the last exon of *At1g11890* (Figure 1A). RT-PCR detection of the fragment before or spanning the T-DNA insertion site indicated that AtSEC22 expression was either very low in some regions (exons 2–4), or could not be detected in some other regions (exons 1–3, 3–6) (Figure 1B), suggesting that *AtSEC22* expression is significantly downregulated in *atsec22–4*. We also generated *AtSEC22* RNAi lines, while it was partially downregulated in RNAi lines (Figure 1C). We then generated an anti-AtSEC22 polyclonal antibody against 1–192 amino acids to detect protein levels. Immunoblot analysis indicated that very little AtSEC22 protein accumulated in *atsec22-4*, and protein levels were partially decreased in *AtSEC22* RNAi lines (Figure 1D). *atsec22-4* exhibited serious developmental defects, including delayed germination and lower germination rate (Supplementary Figures 1A,B), short PR, more adventitious roots (Figures 1E,F), and denser root hairs (Figures 1G,H). Moreover, *atsec22-4* plants had dwarfism (Supplementary Figure 1C). To confirm the causal gene, we performed complementation experiments by generating *pAtSEC22:gAtSEC22*-expressing plants harboring a native promoter-driven *AtSEC22* genomic fragment that was introduced into *atsec22-4* by crossing. The complemented *atsec22-4* plants recovered all phenotypes (Figure 1 and Supplementary Figure 2C). All these results show that *AtSEC22* is the gene responsible for *atsec22-4* phenotypes.

### Cell Morphogenesis Was Perturbed in *atsec22-4*

**FIGURE 2:**
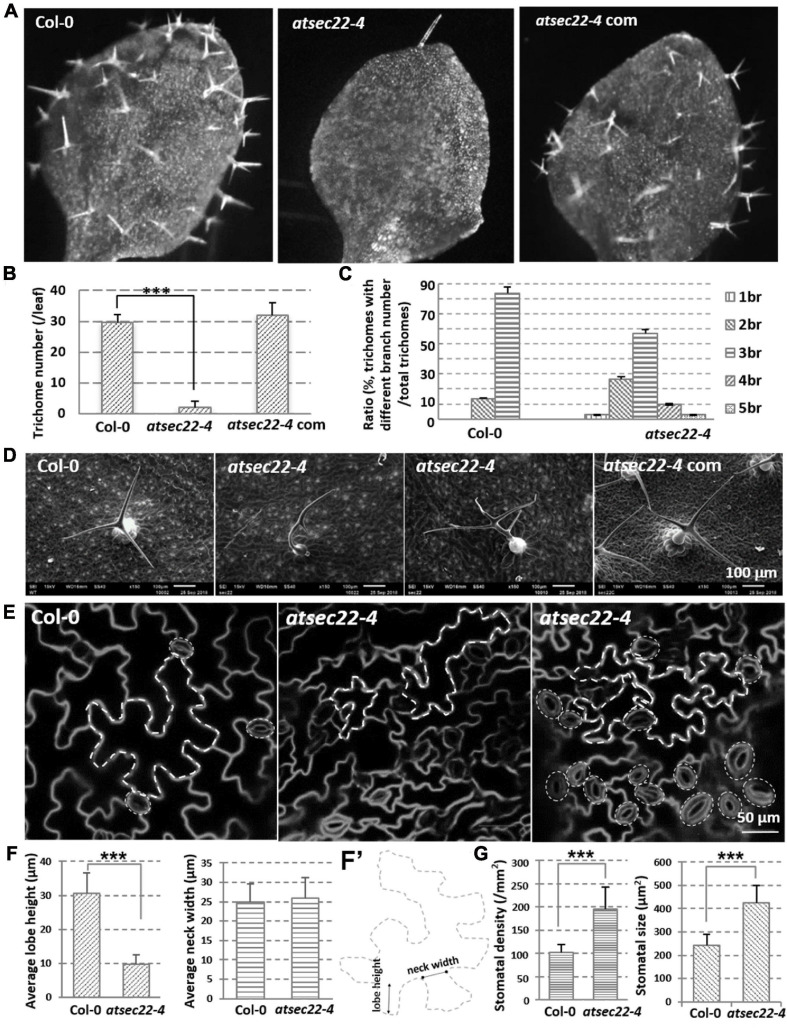
Morphology of epidermal cells was abnormal in *atsec22-4.* **(A)** Images of the first true leaves from 12-day-old seedlings. Images were the same scale. **(B)** Statistics of leaf trichome number in panel (A). *n*_Col–__0_ = 12, *n_*atsec*__22__–__4_* = 20 leaves. ***P < 0.001. Significance was evaluated by Student’s *t* test. **(C)** Statistics of leaf trichomes with different branch number from seven-week-old plants. *n*_Col–__0_ = 66, *n_*atsec*__22__–__4_* = 46 trichomes. br, branch. **(D)** SEM images of 3rd or 4th rosette leaves from seven-week-old plants. **(E)** FM4-64-labeled shape of pavement cells and stomata in the 4th leaves from 14-day-old seedlings. Scale bars are as shown. **(F,F’)** The measurement of lobe length and neck width of pavement cells in the middle part of leaves from 14-day-old seedlings. *n* ≥ 20. Panel **(F’)**, the line with round dots indicates neck width; the line with arrows indicates lobe height. **(G)** Statistics of leaf stomata density and size in the middle part of the first pair true leaves from 12-day-old seedlings. For density statistics, 20 slices of an area of 0.02 mm^2^ were used; for size statistics, *n*_Col–__0_ = 40, *n_*atsec*__22__–__4_* = 50 stomata. ***P < 0.001. Significance was evaluated by Student’s *t* test.

In addition to the developmental defects, the more conspicuous phenotype in *atsec22-4* was that *atsec22-4* had fewer trichomes on the leaves (Figures 2A,B, and Supplementary Figure 2A) and fewer hairs on the stems (Supplementary Figure 2C). SEM and stereomicroscopic observation indicated that trichome morphology in *atsec22-4* was abnormal. In the wild-type, most trichomes (83.3%) had three branches, but in *atsec22-4*, only 56.7% of trichomes had three branches, 26.7% had two branches, and the rest had four, five, or no branches (Figures 2C,D, and Supplementary Figure 2B). In the *AtSEC22* RNAi lines, delayed development (Supplementary Figure 3A), decreased trichome number (Supplementary Figures 3B,D), and altered morphology (Supplementary Figures 3C,E) were also observed. Since the trichome is a specialized cell type originating from epidermal cells, we then examined epidermal cells. The pavement cells adopted jigsaw puzzle-like shapes with interlocking lobes and necks in the wild-type plant. However, in *atsec22-4*, pavement cells exhibited irregular shapes (Figures 2E, dotted lines, Supplementary Figure 2D). We measured the lobe length and neck width of the pavement cells, and found that the lobe length in *atsec22-4* was significantly shorter, while the neck width was not significantly different (Figures 2F,F’). Moreover, an increased number of stomata with disordered distribution and uneven size were observed in *atsec22-4* leaves (Figure 2E, dotted circles, Figure 2G, and Supplementary Figure 2D). These results indicated that cell morphogenesis was perturbed in *atsec22-4.*

### Downregulation of *AtSEC22* Affected Vesicle Trafficking and Integrity of Endomembrane System

**FIGURE 3:**
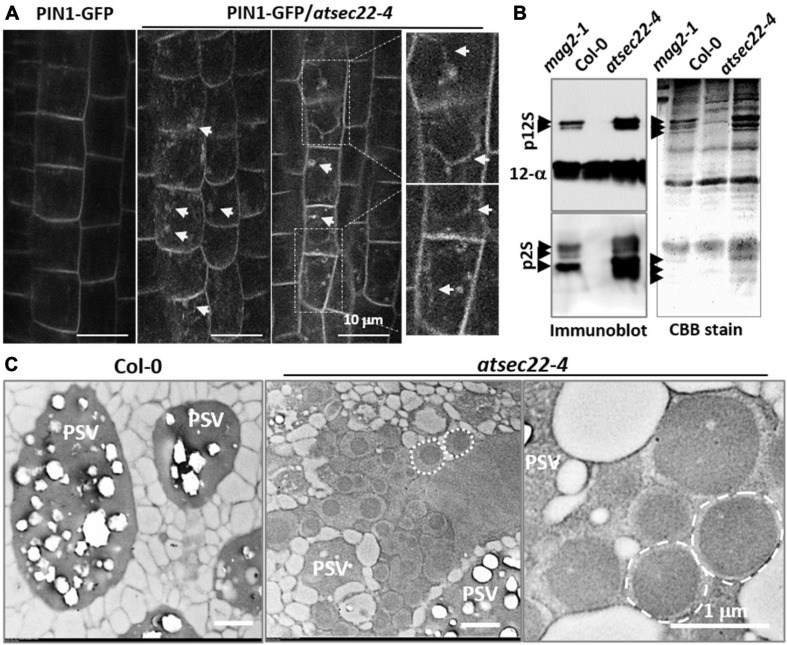
Vesicle trafficking was disturbed in *atsec22-4.* **(A)** PIN1-GFP were internalized in root cells in *atsec22-4* (arrows). Magnified images emphasize multiple localization of PIN1-GFP (arrows). **(B)** Immunoblot of seed proteins with anti-12S globulin and anti-2S albumin antibodies **(left)**, and total seed protein profiles (CBB-stained SDS-PAGE gel) **(right)**. The *atsec22-4* seeds accumulated numerous SSP precursors (arrows). *mag2-1* was used as positive control, Col-0 was used as negative control. p12S, 12S globulin precursors; p2S, 2S albumin precursors; 12S-α, 12S globulin α subunits. Scale bars are as shown. **(C)** TEM images of dry seed cells. Unusual structures (dotted circles) with a high electron-dense core were developed in *atsec22-4* seed cells. PSV, protein storage vacuole.

To investigate the effects on vesicle transport in *atsec22-4*, we introduced the trans-Golgi marker ST-GFP (Boevink et al., 1998), the ER-retention marker GFP-HDEL (Matsushima et al., 2002), and the plasma membrane-targeting marker PIN1-GFP (Benkova et al., 2003), into *atsec22-4* by crossing. Confocal images revealed that Golgi-resident ST-GFP proteins were collapsed and redistributed to the ER, and some ST-GFP aggregates accumulated in the ER tubular intersections in *atsec22-4* (Supplementary Figure 4A), suggesting that ER export was blocked. The GFP-HDEL-visualized ER network exhibited a relatively looser structure with a larger space between the ER tubules in *atsec22-4* (arrows), suggesting that the ER structure was disturbed in the mutant (Supplementary Figure 4B). Moreover, a large amount of intracellular-localized PIN1-GFP was observed in stele cells in *atsec22-4* (Figure 3A, arrows), suggesting disturbed membrane trafficking. We then examined the accumulation of precursors of seed storage proteins (SSPs), which are considered as markers for ER-to-vacuolar transport (Li et al., 2006, 2013; Shimada et al., 2006; Zhao et al., 2018). As shown in Figure 3B, numerous SSP precursors accumulated in *atsec22-4* seeds, indicating that the ER-to-vacuole pathway was blocked.

To investigate the effects of protein accumulation on subcellular structures in the mutant, dry seeds and leaves were examined using TEM analyses. In *atsec22-4* seed cells, numerous unusual structures with a high electron-dense core were developed (dotted circles) (Figure 3C). The unusual structures resembled those in *mag2, mag4, mag5*, and *mip1/2/3* mutants defective in ER export. The structures have been shown to be composed of precursors of 2S albumins and 12S globulins (Li et al., 2006, 2013; Takahashi et al., 2010; Takagi et al., 2013), and the ER chaperons BiP and PDI, implying their ER lumen-localization (Li et al., 2006). These results suggested that ER export was blocked in *atsec22-4*. TEM observations of leaf cells revealed that in *atsec22-4*, the ER network lost most of the tubular structures and produced expanded and fragmented ER sheets (Supplementary Figure 4C, black arrows). Furthermore, the Golgi stacks became smaller with fewer cisternae, indicating that the endomembrane system was collapsed due to a block in vesicle trafficking. The trap of proteins inside the ER usually induces ER stress. Determination of the expression levels of the ER stress-specific marker BiP3 indicated that its transcriptional expression was substantially increased in *atsec22-4* (Supplementary Figure 5A), suggesting serious ER stress. ER stress usually induces the unfolded protein response (UPR) pathway (Sundaram et al., 2018). We examined the expression levels of UPR pathway markers, *IRE1A*, *IRE1B*, and *bZIP60*, and found that *bZIP60* expression was significantly upregulated (Supplementary Figure 5B), suggesting that the UPR pathway was affected in *atsec22-4*. Taken together, these results indicated that vesicle trafficking and integrity of the endomembrane network were affected in *atsec22-4*.

### AtSEC22 Interacted With SNARE Protein AtSYP32

**FIGURE 4:**
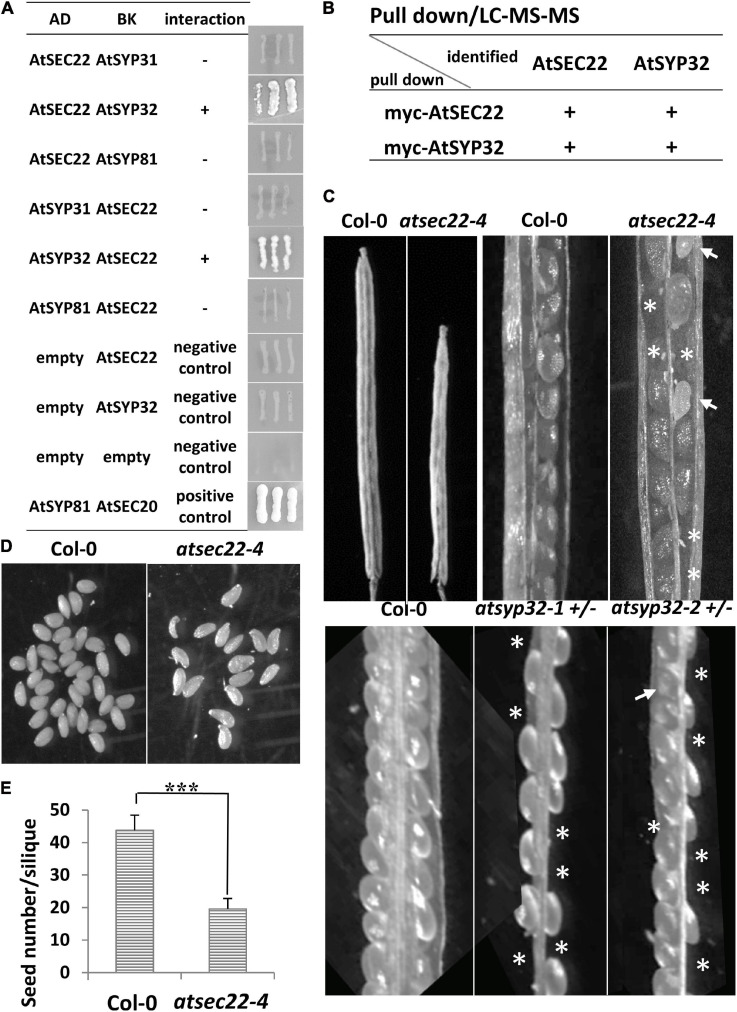
AtSEC22 interacts with Golgi-localized syntaxin AtSYP32. **(A)** AtSEC22 interacts with AtSYP32 rather than with AtSYP31 and AtSYP81. Plasmid combinations are shown. Yeast strain AH109 was transformed with the paired constructs. Transformants were streaked onto SD/_Leu/_Trp/_His/_Ade medium. +, interaction; –, no interaction; AD, pGADT7; BK, pGBKT7. **(B)** Pull down assay using myc-*AtSEC22*-overexpressing plants followed by shotgun LC-MS/MS analysis. As a results, AtSYP32 was identified. Inverse experiments identified AtSEC22. **(C)** *atsec22-4*, *atsyp32-1*±, and *atsyp32-2* ± had smaller siliques and were partially abortive. Asterisks, positions of abortive seeds; arrows, poorly developed seeds. **(D)** Siliques in *atsec22-4* had fewer seeds than those in wild-type. The seeds were from one silique. **(E)** Statistics of panel **(D)**. *n* ≥ 15. ***P < 0.001. Significance was evaluated by Student’s *t* test.

In yeast and mammalian cells, Sec22 mediates anterograde and retrograde transport between the ER and Golgi complex (Flanagan et al., 2015; Li et al., 2015; Zhao et al., 2015). To investigate which transport pathway is regulated by AtSEC22, we performed yeast two-hybrid (Y2H) analysis. Y2H analysis detected an interaction between AtSEC22 and the Golgi-resident syntaxin AtSYP32 (*At3g243500*), but no interaction of AtSEC22 with AtSYP31 or the ER-localized syntaxin AtSYP81 (Figure 4A). We further performed pull-down analysis using TAP (myc)-tagged *AtSEC22*-overexpressing plants followed by shotgun LC-MS/MS analysis, and identified AtSYP32. To confirm this interaction, we generated myc-tagged *AtSYP32*-over-expressing plants and performed pull-down analysis followed by shotgun LC-MS/MS analysis. As expected, AtSEC22 was identified (Figure 4B), suggesting that AtSEC22 interacts with AtSYP32 in plant cells and might regulate anterograde transport. We then isolated the *atsyp32-1* and *atsyp32-2* mutants and found that they were homozygously lethal. This sterile phenotype was similar to the homozygous mutant, *atsec22-1*, which has pollen lethality (El-Kasmi et al., 2011), suggesting that AtSEC22 and AtSYP32 are essential for reproductive development. The heterozygotes, *atsyp32-1*± and *atsyp32-2*±, had shorter siliques and were partially abortive; these phenotypes were similar to those of *atsec22-4*, the downregulation mutant (Figures 4C–E).

### Cytoskeleton Organization Is Disturbed in *atsec22-4*

**FIGURE 5:**
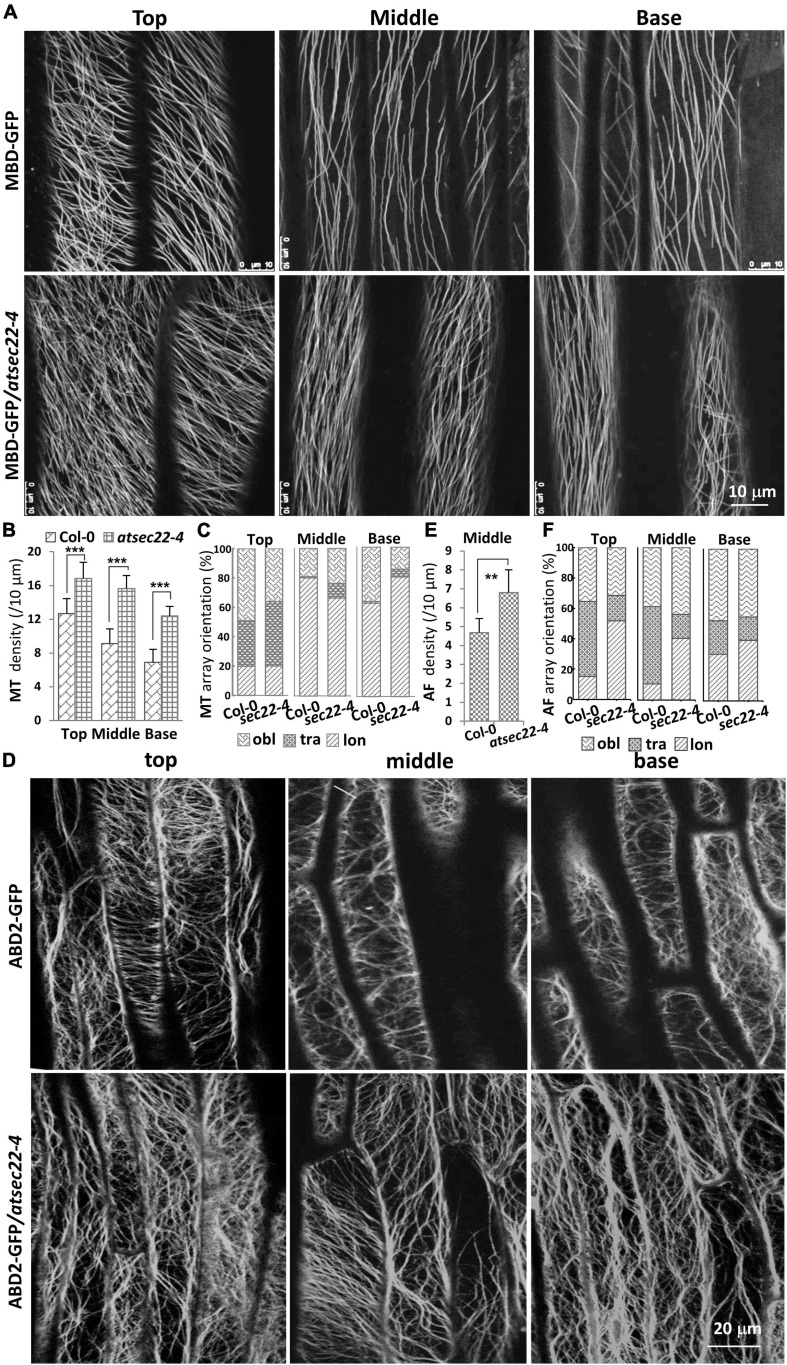
Cytoskeleton organization was disturbed in *atsec22-4.* **(A)** Confocal images of MBD-GFP-visualized cortical MTs in the top, middle, and basal regions of hypocotyls from five-day-old seedlings grown in the dark. **(B)** Statistics of cortical MT density in panel **(A)**. **(C)** Statistics of cortical MT array orientations in (A). **(D)** Confocal images of ABD2-GFP-labeled AFs in the top, middle, and basal regions of hypocotyls. **(E)** Statistics of AF density in middle region of hypocotyls (D). **(F)** Statistics of AF array orientations in panel **(D)**. lon, longitudinal; tra, transverse; obl, oblique. *n* ≥ 10 cells. Scale bars are as shown. **P < 0.01, ***P < 0.001. Significance was evaluated by Student’s *t* test.

It has been reported that the cytoskeleton controls trichome morphogenesis, MTs determine trichome formation and branch number, and AFs control trichome morphology (Kim et al., 2002; Mathur, 2005; Szymanski, 2005; Sambade et al., 2014). Therefore, we introduced the MT markers, MBD-GFP and TUA6-GFP, and the AF marker ABD2-GFP into *atsec22-4* by crossing. Confocal microscope analysis revealed that the organization of cortical MTs and AFs was altered in *atsec22-4*. In hypocotyl from five-day-old seedlings grown in the dark, MBD-GFP-visualized cortical MTs in *atsec22-4* were thinner and denser than that in the wild-type, in the top, middle, and base parts of hypocotyl (Figures 5A,B). Furthermore, the MT array orientation was also altered (Figure 5C). ABD2-GFP-visualized AFs maintained transverse or oblique alignment in most epidermal cells in wild-type hypocotyl. However, in *atsec22-4* hypocotyl, AF arrays became more extensive (Figures 5D,E), and longitudinally aligned AFs and thicker actin bundles were more frequently observed (Figure 5F). All these observations suggested that cytoskeleton organization was disturbed in *atsec22-4.*

### AtSEC22 Modulates Cytoskeleton Stability

**FIGURE 6:**
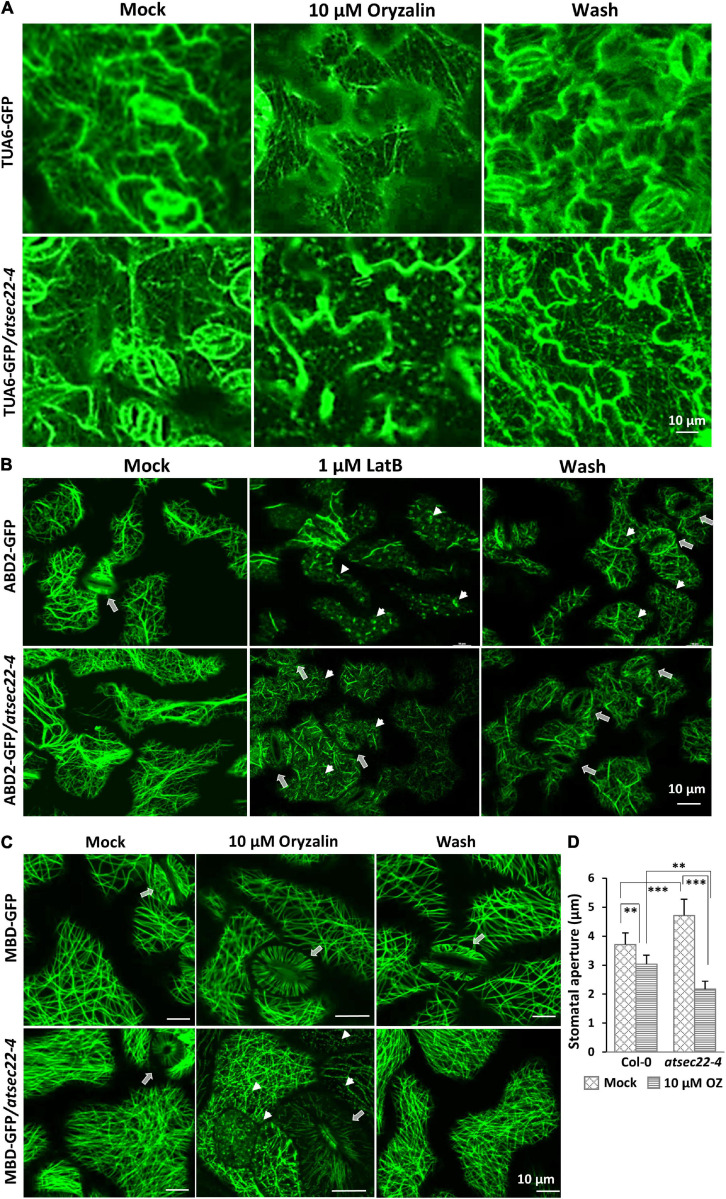
Cytoskeleton stability was perturbed in *atsec22-4.* **(A)** Cortical MTs in *atsec22-4* were sensitive to oryzalin treatment. TUA6-GFP-visualized cortical MTs in leaves from 12-day-old seedlings were treated with 10 μM of oryzalin for 20 min, and followed by a 2 h wash. **(B)** Cortical AFs in *atsec22-4* were resistant to LatB treatment. ABD2-GFP-visualized cortical AFs in leaves from 12-day-old seedlings were treated with 1 μM of LatB for 20 min, and followed by a 2 h wash. Arrows, stomata; arrow heads, depolymerized AFs. **(C)** Stomata movement was abnormal in *atsec22-4*. MBD-labeled cortical MTs in leaves from 12-day-old seedlings were treated with 10 μM of oryzalin for 20 min, and followed by a 2 h wash. Arrows, stomata; arrow heads, depolymerized MTs. **(D)** Statistics of stomata aperture in panel **(C)**. *n* ≥ 30. Scale bars are as shown. **P < 0.01, ***P < 0.001. Significance was evaluated by Student’s *t* test.

We further examined the stability of MTs and AFs using the microtubule-disrupting drug, oryzalin, and the actin polymerization inhibitor, LatB, respectively. After 10 min of treatment with 10 μM of oryzalin to hypocotyls and 20 min to the leaves, TUA6-GFP-visualized cortical MTs in wild-type leaf cells (Figure 6A) and MBD-GFP-visualized MTs (Supplementary Figure 6A) in wild-type hypocotyl cells just began to be depolymerized, appearing as GFP-labeled dots. However, in most of the *atsec22-4* leaf and hypocotyl cells, cortical MTs were almost completely depolymerized. After 2 h of washing, the alignment of cortical MTs in the wild-type was recovered, whereas that in *atsec22-4* was significantly delayed. A large number of dots remained, but they were organized along the MT arrays (Figure 6A and Supplementary Figure 6A). As for AFs, after 20 min treatment with 1 μM of LatB, most of the ABD2-GFP-visualized AFs in wild-type leaf cells lost their linear alignment, whereas AFs in *atsec22-4* showed significant delay (Figure 6B). These results suggest that AtSEC22 is vital for cytoskeleton stability.

Stomatal movement is closely correlated with AF and MT dynamics (Lemichez et al., 2001; Zhang et al., 2007; Zhao et al., 2011; Eisinger W. R. et al., 2012). In wild-type, stomata are usually open with proper apertures. However, in *atsec22-4*, the stomata seemed open with a relatively wider and uneven aperture (Figure 2E and Supplementary Figure 2D). Following treatment with 10 μM of oryzalin for 20 min, stomata in *atsec22-4* closed with a larger range than those in the wild-type (Figures 6C,D). The sensitivity of stomata to oryzalin further indicated altered cytoskeleton stability in *atsec22-4*.

**FIGURE 7:**
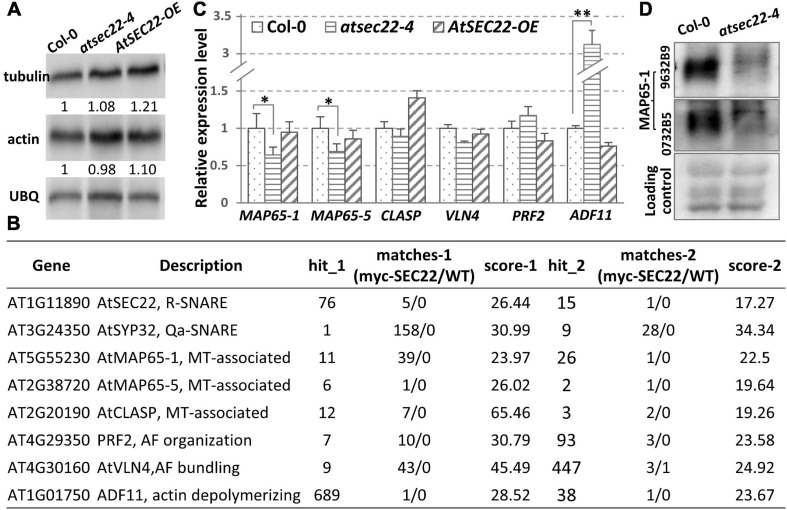
Transcription levels of cytoskeleton-associated proteins were affected in *atsec22-4.* **(A)** Immunoblot analysis using ten-day-old seedlings with anti-actin and anti-tubulin antibodies. Ubiquitin (UBQ) was used as endogenous control. **(B)** LC-MS/MS identified cytoskeleton-regulators from pull down assay using myc-*AtSEC22*-overexpressing plants. Hit_1, peptide_hit_number_1; score-1, peptide score-1; WT, Col-0. **(C)** Transcriptional levels of cytoskeleton-regulator genes. Two independent experiments for each sample, three replicates for each experiment. *P < 0.05, **P < 0.01. Significance was evaluated by Student’s *t* test. **(D)** Protein levels of MAP65-1 were determined with two anti-MAP65-1 antibodies against two different peptides of MAP65-1 using ten-day-old seedlings.

To determine the protein levels of tubulin and actin in *atsec22-4*, we performed immunoblot analysis. The results indicated no significant change in tubulin and actin levels in *atsec22-4* (Figure 7A), indicating that the abnormalities of the cytoskeleton were not due to the cytoskeletal component proteins. To determine the reasons for defects in cytoskeleton dynamics, we re-analyzed the pull down-LC-MS/MS results and identified some cytoskeleton-associated proteins (Figure 7B). We found that the expression levels of *AtMAP65-1*, *AtMAP65-5*, and *ADF11* were significantly altered (Figure 7C). We further examined the protein level of MAP65-1, an MT-associated protein required for MT depolymerization and reorganization (Smertenko et al., 2004; Gaillard et al., 2008; Lucas et al., 2011; Zhou et al., 2017), using two antibodies against two different parts of the MAP65-1 protein. The results indicated that the protein levels of MAP65-1 were also dramatically decreased in *atsec22-4* (Figure 7D), suggesting that the stability of the cytoskeleton regulators was affected.

## Discussion

### AtSEC22 Regulates Secretion Pathway

SNARE proteins contribute to efficient membrane fusion. In yeast and mammals, Sec22 localizes to the ER and Golgi and regulates anterograde and retrograde transport (Flanagan et al., 2015; Li et al., 2015; Zhao et al., 2015), and Sec22 is recruited to the COPII vesicles by Sec23/24 (Mancias and Goldberg, 2007). Plant SEC22 has been reported to function in the early secretory pathway and is essential for the integrity of the ER network and Golgi complex (Chatre et al., 2005; El-Kasmi et al., 2011). In this study, we found that AtSEC22 interacts with the Golgi-syntaxin AtSYP32. Since AtSYP32 is predicted as a Qa-SNARE localized in cis-Golgi, it is quite possible that AtSEC22 cooperates with AtSYP32 to regulate ER-Golgi transport. However, even if AtSYP32 is usually used as a Golgi marker, its biological function has not been reported so far. Interestingly, interactome analysis of Qa-SNARE proteins revealed that cytoskeleton components were enriched in the SYP3 (SYP31 and SYP32) group (Fujiwara et al., 2014), suggesting potential relations between the cytoskeleton and AtSYP32.

A large amount of intracellular-localized PIN1-GFP was observed in stele cells in *atsec22-4*. That it resulted from the blocked ER-Golgi trafficking or another transport pathway affected in *atsec22-4* needs further investigation to clarify. Because the ER and Golgi morphology were disturbed seriously in *atsec22-4*, combined with the fact that the Golgi apparatus is the hub for intracellular vesicle trafficking, it is possible that the secretion pathway and potentially also the recycling pathway were defective in *atsec22-4*. Further investigation is needed to clarify these points.

### AtSEC22 Is Involved in Cell Morphogenesis and Plant Development

AtSEC22 has previously been reported to regulate gametophyte development (El-Kasmi et al., 2011), our study found that *atsec22-4* exhibited serious developmental and reproduction defects. The impaired morphogenesis in *atsec22-4* were due to disrupted organization and stability of the MT and AF cytoskeleton. And the reproduction defects could have resulted from dysfunction of the spindle, which is composed of MTs. These abnormalities might be due to dis-homeostasis of the MT- and AF-associated proteins. These regulators might be synthesized in cytoplasm, however, some of them need modification for activation. Such as the MAP65-1 phosphorylation form which is required for MT depolymerization and reorganization. Its phosphorylation is regulated by Aurora-, cyclin dependent kinases (CDK)-, and MAPK-dependent pathways for different functions (Smertenko et al., 2004, 2006; Sasabe and Machida, 2012; Smékalová et al., 2014; Boruc et al., 2017; Zhou et al., 2017; Vavrdová et al., 2019). These enzymes are usually synthesized in the ER then delivered to the Golgi apparatus. It is quite possible that the export of them from the ER was affected and subsequently disturbed cytoskeleton regulators’ modification in *atsec22-4*. Reduction of MAP65-1 might be one of the triggers for MT disorganization and/or depolymerization. It has been reported that the morphology of leaf pavement cells in *AtMAP65-1*-overexpressing plants was significantly altered, and the lobe length was obviously decreased resulting in a smooth cell shape. Whereas the *atmap65-1* mutant had denser root hairs, and some of them seemed swollen. Furthermore, the alignment of MTs in both the mutant and overexpression lines was altered (Chen, 2009). MAP65-1 was recently reported to inhibit katanin to bind to MT bundles, thus protecting them from severing (Burkart and Dixit, 2019), suggesting that MAP65-1 is essential for MTs stability. Moreover, MAP65-1 and its homolog MAP65-5 have spatiotemporal colocalization, they are concentrated at the midzones of the spindle during anaphase B and the phragmoplast (Smertenko et al., 2008), and they also colocalize with cortical MTs (van Damme et al., 2004; Lucas et al., 2011), suggesting their functional redundancy. Taken together, AtSEC22 regulates plant morphogenesis and development by controlling cytoskeleton dynamics.

Largely, plant development relies on the pattern of cell development. It is well known that cytoskeleton dynamics are vital for vesicle trafficking. Our findings provide evidence that cytoskeleton dynamics are essential for ER-Golgi trafficking. The impaired alignment of the cytoskeleton in *atsec22-4* strongly suggested the dependence of AtSEC22-mediated vesicle trafficking on the cortical MTs and AFs. On the other hand, the cytoskeleton organization and stability are dependent on vesicle trafficking which probably control homeostasis of MT-/AF-associated regulators. Extensive in-depth research is expected to clarify the crosstalk. In conclusion, vesicle trafficking and cytoskeleton are closely interdependent.

## Data Availability Statement

The original contributions presented in the study are included in the article/Supplementary Material, further inquiries can be directed to the corresponding author.

## Author Contributions

LL and XL conceived the project. LL, LG, and XL designed the experiments. LG and SY generated all material used in this study (cloning, transformations, transgenic plants, and crosses). LG, SL, YL, YY, YQL, and TW were involved in the confocal observation, trichome statistics, immunoblot, plant handling, and post-acquisition image analysis. GQ performed the LC-MS/MS. HW generated the TSM images of seeds. J-KZ, YW, XF, and ZW contributed the reagents, materials, and the analytical platform. LL and LG drafted the manuscript. All authors commented on the manuscript.

## Conflict of Interest

The authors declare that the research was conducted in the absence of any commercial or financial relationships that could be construed as a potential conflict of interest.

## Footnotes

1 https://www.miltenyibiotec.com/en/

2 https://www.arabidopsis.org/

## Supplementary Material

Supplementary Figure 1 Development of *atsec22-4* was delayed. **(A)** Germination of *atsec22-4* was delayed. Germination ratio was quantified using three-day-old seedlings. **(B)** Statistics of panel **(A)**. *n* ≥ 30. Three independent experiments per sample. ^∗∗^*P* < 0.01. **(C)** *atsec22-4* plants were dwarf.

Supplementary Figure 2 Morphogenesis was disturbed in *atsec22-4*. **(A)** There were less leaf trichomes in 40-day-old plants in *atsec22-4*. **(B)** Morphology of trichomes in *atsec22-4* were altered. Trichomes were from the 3rd/4th rosette leaves in seven-week-old plants. **(C)** There were less hairs on stems in *atsec22-4*. **(D)** Morphology of pavement cells and stomata were altered in *atsec22-4*.

Supplementary Figure 3 Plant development and morphogenesis were disturbed in *AtSEC22* RNAi lines. **(A)** Development of *AtSEC22* RNAi plant was delayed. **(B)** Trichome number was decreased in *AtSEC22* RNAi leaves. **(C)** Trichome morphology was altered in *AtSEC22* RNAi lines. **(D)** Statistics of trichome number of panel **(B)**. *n* ≥ 10 leaves. ^∗∗^*P* < 0.01. **(E)** Statistics of leaf trichomes with different branch number. *n*_Col–0_ = 20, *n*_RNAi_ = 30.

Supplementary Figure 4 Vesicle trafficking was disturbed in *atsec22-4*. in *atsec22-4.* **(A)** Confocal images of ST-GFP-visualized Golgi apparatus in root cells. ST-GFP was collapsed from the Golgi and redistributed in the ER in *atsec22-4* (arrows). **(B)** Confocal images of GFP-HDEL-visualized ER network in pavement cells. The ER exhibited relatively looser structure with larger space between the ER tubules in *atsec22-4* (arrows). **(C)** TEM images of leaf cells from 21-day-old plants. In *atsec22-4*, the ER tubules became expanded and fragmented (black arrows), and the Golgi stacks became smaller in width and consisted of less cisternae.

Supplementary Figure 5 ER stress was induced in *atsec22-4*. **(A)** Expression level of *BiP3* was detected by RT-PCR. **(B)** Expression levels of *IRE1A*, *IRE1B* and *bZIP60* were detected by RT-qPCR.

Supplementary Figure 6 Microtubule stability was perturbed in *atsec22-4*. Confocal images of MBD-GFP-visualized cortical MTs in hypocotyls treated with 10 μM of oryzalin for 10 min **(A)**, and followed by a 2 h wash **(B)**. Arrows in panel **(A)**, depolymerized MTs; arrows in panel **(B)**, MTs started to line up.

Supplementary Table 1 List of primers used in this study.

Supplementary Table 2 Original data of LC-MS/MS.
