# Supplementary material for: AtSEC22 Regulates Cell Morphogenesis via Affecting Cytoskeleton Organization and Stabilities
Source: Front Plant Sci. 2021 Jun 4;12:635732. doi: 10.3389/fpls.2021.635732 (PMC8211912; doi:10.3389/fpls.2021.635732)
Supplement: Supplementary Figure 1 — Development of atsec22-4 was delayed. (A) Germination of atsec22-4 was delayed. Germination ratio was quantified using three-day-old seedlings. (B) Statistics of panel (A). n ≥ 30. Three independent experiments per sample. ∗∗P < 0.01. (C) atsec22-4 plants were dwarf. [file Data_Sheet_1.pdf]

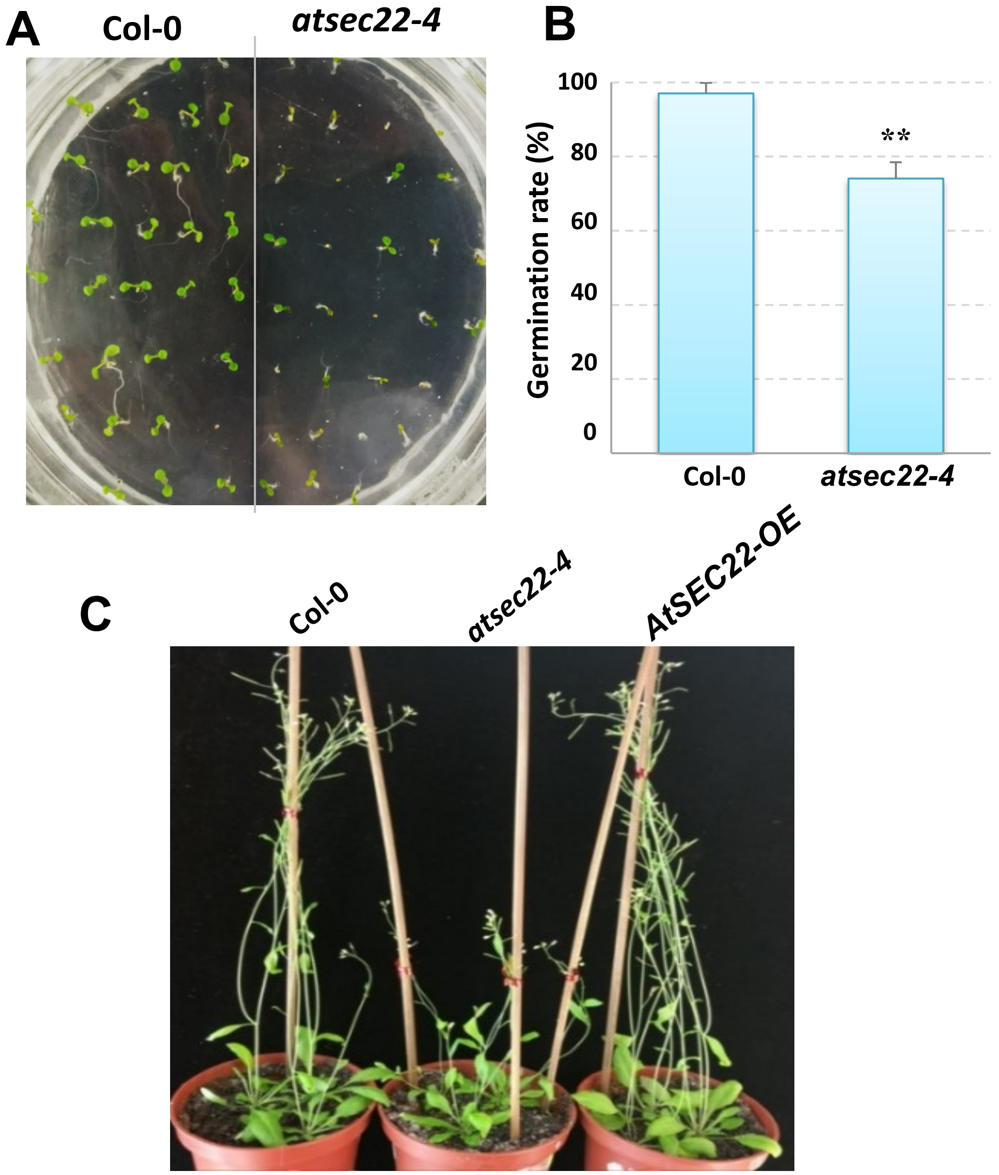

Figure S1. Development of *atsec22-4* was delayed.

(A) Germination of *atsec22-4* was delayed. Germination ratio was quantified using three-day-old seedlings.

(B) Statistics of (A). n≥30. Three independent experiments per sample. **P < 0.01.

(C) *atsec22-4* plants were dwarf.

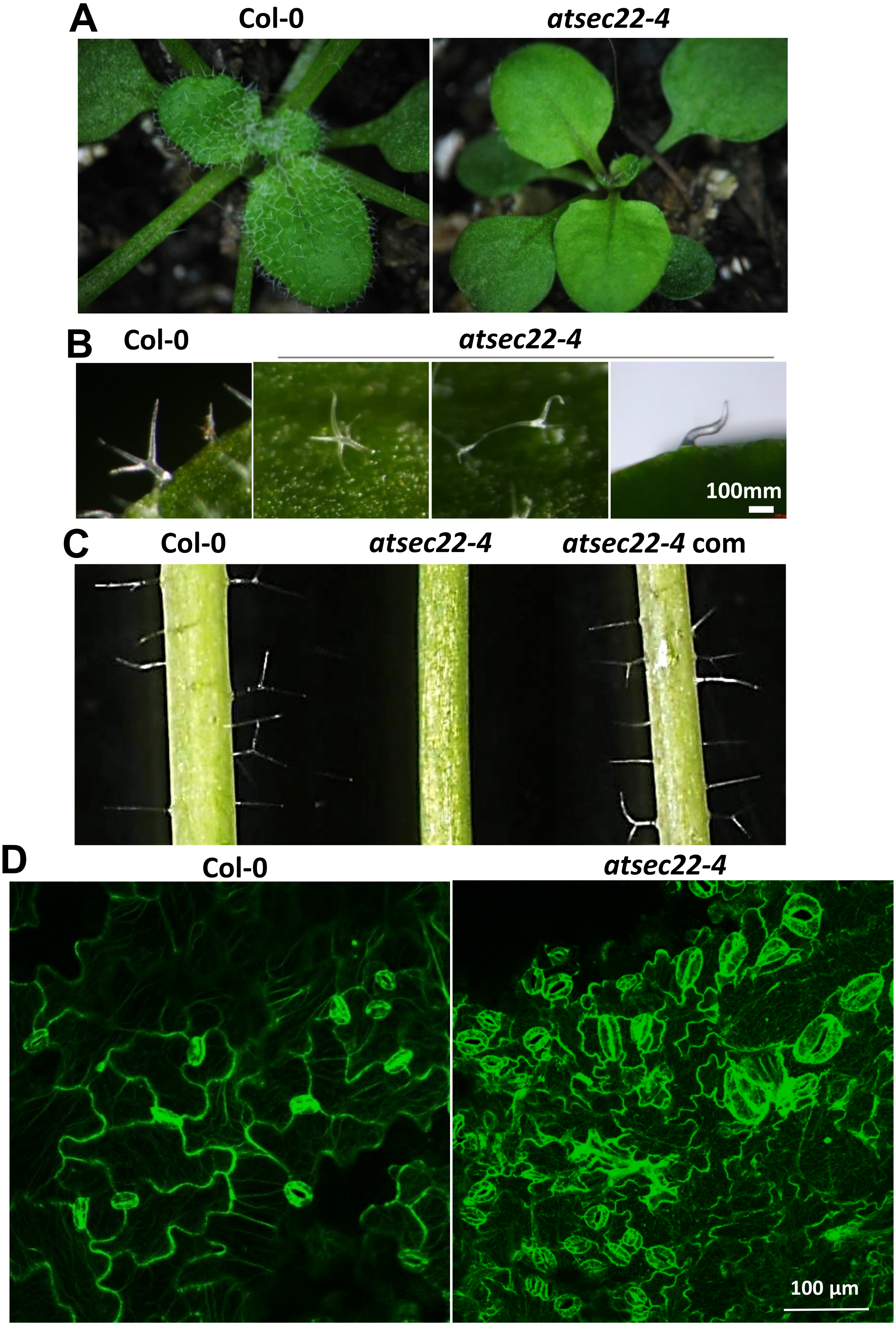

Figure S2 | Morphogenesis was disturbed in *atsec22-4*.
(A) There were less leaf trichomes in *atsec22-4* 40-day-old plants. .
(B) Morphology of leaf trichomes in *atsec22-4* were altered. Trichomes were from the 3rd/4th rosette leaves in seven-week-old plants.
(C) There were less hairs on stems in *atsec22-4* .
(D) Morphology of pavement cells and stomata were altered in *atsec22-4*.

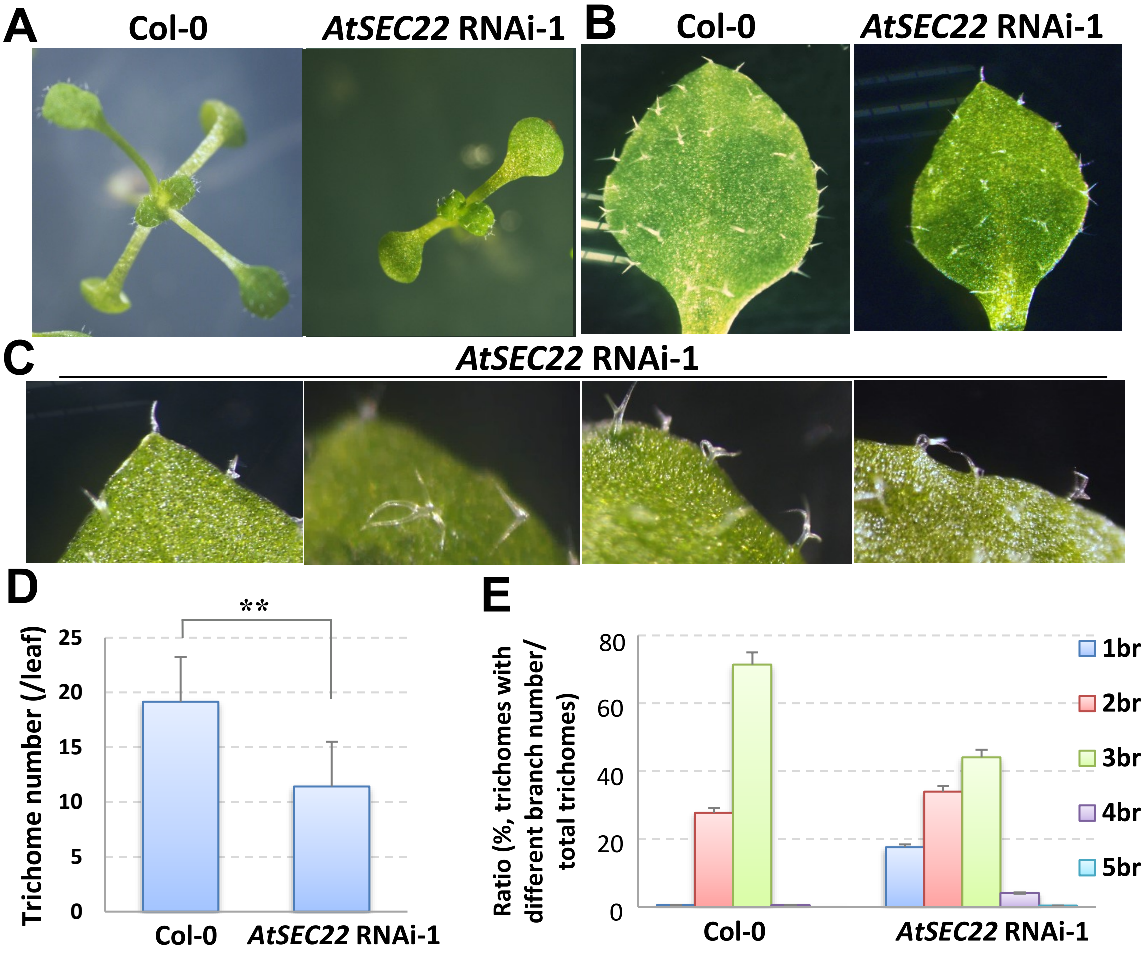

Figure S3. Development and morphogenesis were disturbed in *AtSEC22* RNAi lines.

(A) Development of *AtSEC22 RNAi* plant was delayed.

(B) Trichome number was decreased in *AtSEC22 RNAi* leaves.

(C) Trichome morphology was altered in *AtSEC22 RNAi* lines.

(D) Statistics of trichome number of (**B**). n≥10 leaves. **$P < 0.01$.

(E) Statistics of leaf trichomes with different branch number in *AtSEC22* RNAi lines. n_{Col-0}=20, n_{RNAi}=30.

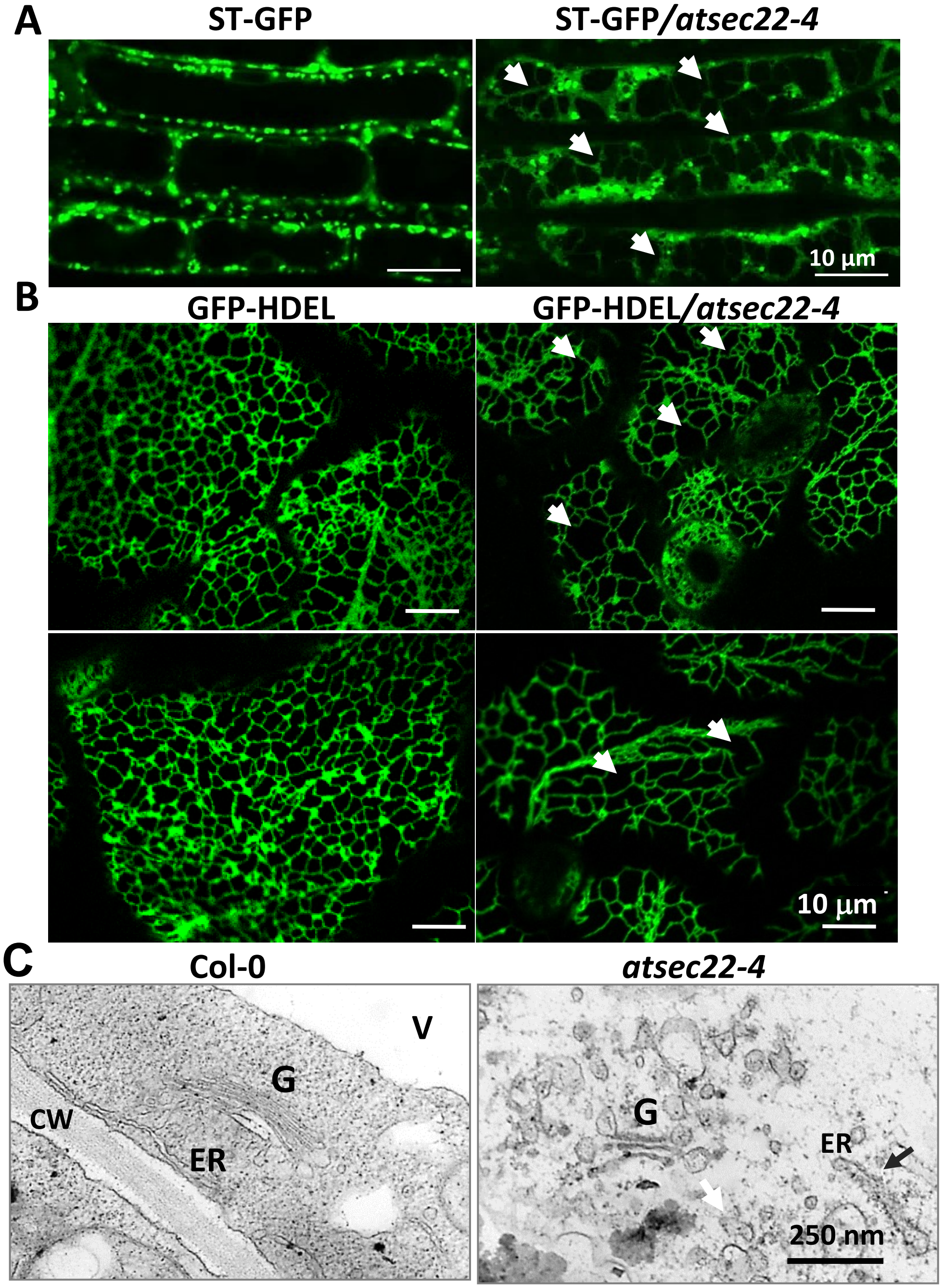

Figure S4. Vesicle trafficking was disturbed in *atsec22-4*.

(A) Confocal images of ST-GFP-visualized Golgi apparatus in root cells. ST-GFP was collapsed from the Golgi and redistributed in the ER in *atsec22-4* (arrows).

(B) Confocal images of GFP-HDEL-visualized ER network in pavement cells. The ER exhibited relatively looser structure with larger space between the ER tubules in *atsec22-4* (arrows).

(C) TEM images of leaf cells from 21-day-old plants. In *atsec22-4*, the ER tubules became expanded and fragmented (black arrows), and the Golgi stacks became smaller with less cisternae. V, vacuole; CW, cell wall; G, Golgi apparatus; ER, endoplasmic reticulum. Scale bars are as shown.

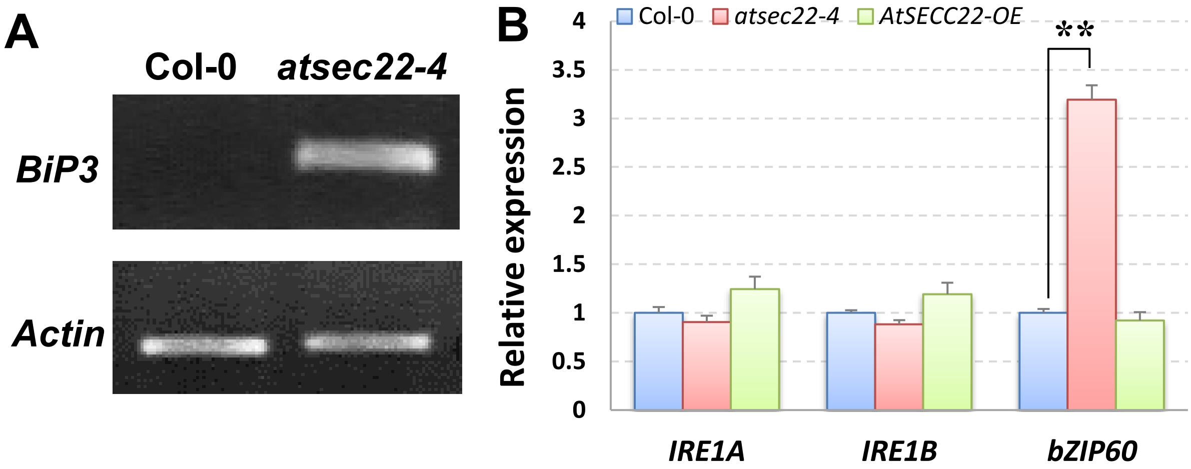

Figure S5. ER stress was induced in *atsec22-4*.

(A) Expression level of *BiP3* was detected by RT-PCR.

(B) Expression levels of *IRE1A*, *IRE1B* and *bZIP60* were detected by RT-qPCR.

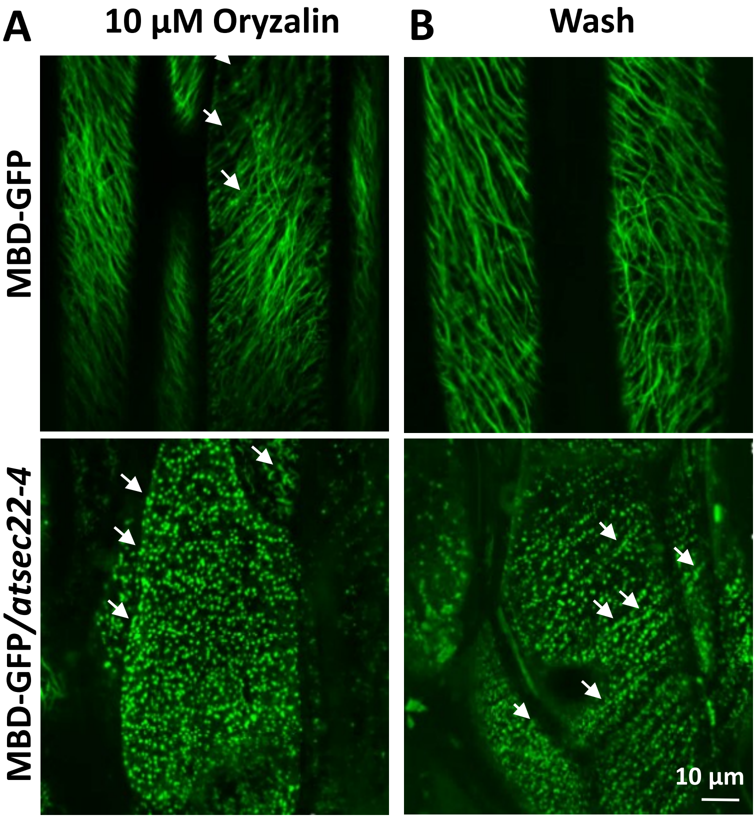

Figure S6. Microtubule stability was perturbed in *atsec22-4*.
Confocal images of MBD-GFP-visualized cortical MTs in hypocotyls treated with 10 μM of oryzalin for 10 min (A), and followed by a 2 h wash (B). Arrows in (A), depolymerized MTs; Arrows in (B), MTs started to line up.
