# Supplementary material for: AtSEC22 Regulates Cell Morphogenesis via Affecting Cytoskeleton Organization and Stabilities
Source: Front Plant Sci. 2021 Jun 4;12:635732. doi: 10.3389/fpls.2021.635732 (PMC8211912; doi:10.3389/fpls.2021.635732)
Supplement: Supplementary Table 1 — List of primers used in this study. [file Table_1.DOCX]

**Supplementary Table 1.** List of primers used in this study.

| **Name** | **Sequence 5’-3’** | **Name** | **Sequence 5’-3’** |
| --- | --- | --- | --- |
| ***Sec22TOPO-F*** | CACCATGGTGAAAATGACATTGAT | ***BiP3RT-F*** | CCAGGGGAGTGCCACAGAT |
| ***Sec22TOPO-R*** | TCAAGCCTGACGGTTCAAAT | ***BiP3RT-R*** | GTAAACCGACTTAATAACCGGGTC |
| ***Sec22antib-R*** | CTAAAGCCTGACGGTTCAAATCTTTAG | ***Actin2-F*** | AGAGATTCAGATGCCCAGAAGTCTTGTT |
| ***Sec22RNAi-F*** | CACCGCTCTTACCCAAAGAAACTC | ***Actin2-R*** | AACGATTCCTGGACCTGCCTCATCATACTC |
| ***Sec22RNAi-R*** | AAGACTACACCGAACACAAT | ***IRE1A-F*** | AACTTGTTGTTGATCGTGATGG |
| ***Sec22comple-F*** | AAAAAGCAGGCTAAATGGTGAAAATGACATTG | ***IRE1A-R*** | TTAGCAGGAGCCTGATATAACG |
| ***Sec22comple-R*** | AGAAAGCTGGGTATTACCATAGCTTGTTCTTG | ***IRE1B-F*** | CGAAAAGGAAAACGCGTTAATG |
| ***Sec22NdeI-F*** | CGGACGcatatgATGGTGAAAATGACATTG | ***IRE1B-R*** | CAATCCTATCCGTCGATGTGTA |
| ***Sec22BamHI-R*** | AGCACTggatccTTACCATAGCTTGTTCTT | ***bZIP60-F*** | CGTTACTGTTTGCAAAAGGGTA |
| ***SYP31NdeI-F*** | CACCATGGGCTCGACGTTCAGAGA | ***bZIP60-R*** | GGCAAATGAAGTTTACTCCCAG |
| ***SYP31BamHI-R*** | TTATGACGAAATTCGAGTGAGAT | ***MAP65-1-F*** | GAGCTTCTTCAAACCTTGTCAG |
| ***SYP32NdeI-F*** | GGAATTCcatatgATGTCGGCAAGGCATGGG | ***MAP65-1-R*** | TCCTGAAGACTTATCCGGAATG |
| ***SYP32BamHI-R*** | CGggatccTTATGCCACGAAGAAGAGG | ***MAP65-5-F*** | AAACTCGTAAGTTCAGAGCTGA |
| ***Sec22Prom-F*** | GTTTAAACACTTTCAGCAAATGAACTTAAG | ***MAP65-5-R*** | CTTCTTTCTTCTTGGCAAACGA |
| ***Sec22Prom-R*** | GGTACCCTTTGCCTTATTACTTCTCCAGTA | ***ATCLASP-F*** | CTTTCAGGGAATCAAACGCTAG |
| ***Sec22G-F*** | GGCGCGCCTATGGTGAAAATGACAT | ***ATCLASP-R*** | CAGACATTCTTCCCCCTACATT |
| ***Sec22G-R*** | TTAATTAATTTTACTTTAATTGTGGTTCTA | ***ATPRF2-F*** | ATCATACGTCGATGACCATCTC |
| ***SEC22-RT-F1*** | GAGAAGTCGCGAGAAGATGG | ***ATPRF2-R*** | TTGGACAACCATGTACTTCTCA |
| ***SEC22-RT-R1*** | TCCAGGTATTGGAAAGCGAGTTTCT | ***ATVLN4-F*** | GCACAAAAGGAAGGTTCAGAAT |
| ***SEC22-RT-F2*** | TGGACGTGACTTACCGGATT | ***ATVLN4-R*** | TACAAGAGAACAAGTGAGGGTC |
| ***SEC22-RT-R2*** | CTAAGACTTCTTGCACATTCCGGGT | ***ADF11-F*** | CTGCCAGAAGAGCAAAATCTTT |
| ***SEC22-RT-F3*** | CATAGAAGGACGTGTTTGCT | ***ADF11-R*** | CTGTTGCTTGCAATTCAACTTG |
| ***SEC22-RT-R3*** | GAGGTCGACATATGTCTGGA | ***sec22-4-LP*** | GCCTAACATTGAAACAGCTGC |
| ***LB1*** | GCCTTTTCAGAAATGGATAAATAGCCTTGCTTCC | ***sec22-4-RP*** | TAGATTTGGCTGGTCCACAAG |
